# Pattern Recognition via PCNN and Tsallis Entropy

**DOI:** 10.3390/s8117518

**Published:** 2008-11-25

**Authors:** YuDong Zhang, LeNan Wu

**Affiliations:** School of Information Science and Engineering, Southeast University, P.R. China; E-Mail: wuln@seu.edu.cn

**Keywords:** Pattern recognition, feature extraction, pulse coupled neural network, Tsallis entropy

## Abstract

In this paper a novel feature extraction method for image processing via PCNN and Tsallis entropy is presented. We describe the mathematical model of the PCNN and the basic concept of Tsallis entropy in order to find a recognition method for isolated objects. Experiments show that the novel feature is translation and scale independent, while rotation independence is a bit weak at diagonal angles of 45° and 135°. Parameters of the application on face recognition are acquired by bacterial chemotaxis optimization (BCO), and the highest classification rate is 72.5%, which demonstrates its acceptable performance and potential value.

## 1. Introduction

Face recognition is a hot problem in image processing [1]. It has many potential applications, for example, for security systems, man-machine interfaces, and searches of video database or the WWW. Therefore, many researchers are actively working in this filed and many face recognition methods are proposed.

Since face image data are usually high-dimensional and large-scale, it is crucial to design an effective feature extraction method. Researchers have developed many algorithms, such as the Eigenface method [2], the Fisherface method [3], the direct LDA method [4], the uncorrelated optimal discrimination vector (UODV) method [5], the Kernel PCA method [6], *etc.*

However, such models are either subject to problems determined by geometric transforms (scaling, translation or rotation) or to high computational complexity [7]. Moreover, it is known that parallel processing could solve computational complexity but in order to take advantage of it we need parallelizable models [8].

Neural networks (NN) have been widely employed in face recognition applications. It is feasible for classification and results in similar or higher accuracies from fewer training samples. It has some advantages over traditional classifiers due to two important characteristics: their non-parametric nature and the non-Gaussian distribution assumption.

Pulse coupled neural network (PCNN) is called the 3^rd^ generation NN since it has the following advantages: (i) global optimal approximation characteristic and favorable classification capability; (ii) rapid convergence of learning procedure; (iii) an optimal network to accomplish the mapping function in the feed-forward; (iv) no need to pre-train. Hence, in this article, we present a novel face recognition approach based on PCNN.

The structure of this article is as follows. Section 2 introduces the architecture of our proposed novel model. Section 3 gives a brief overview on PCNN. Section 4 discusses how to obtain TNF by PCNN. Section 5 introduces the Tsallis entropy. Section 6 brings forward the MLP. Section 7 is the experiment which checks the Translation, Scaling, and Rotation Independence of our proposed feature. Section 8 is the face recognition system. This proposed method can achieve as high as 72.5% classification rate on a given database. Finally Section 9 concludes this paper.

## 2. Architecture of the Model

**Figure 1.**
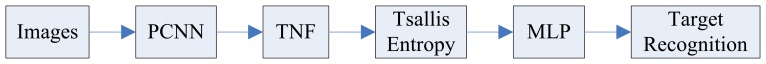
The architecture of the recognition system.

The model proposed in this article is based on three modules: the PCNN, the Tsallis entropy, and the MLP classifier (Figure 1). Information flow is feed-forward but there are also lateral interactions between the PCNN.

Pulse coupled neural network (PCNN) is a result of research on artificial neuron model that was capable of emulating the behavior of cortical neurons observed in the visual cortices of animal [9]. According to the phenomena of synchronous pulse burst in the cat visual cortex, Eckhorn developed the linking field network. Since it does not need to pre-train, and inherits the advantages of artificial neural network (ANN), PCNN has been used for various applications, such as image feature extraction [10], and image segmentation [11], *etc.*

Total number of firing (TNF) is an important parameter obtained in PCNN. It is almost unique and has strong anti-noise ability. How to measure the information obtained in TNF? Communication systems are less well defined in PCNN. Thus traditional Shannon entropy is not fit for measuring. R. Sneddon discussed the Tsallis entropy of neurons and found it is more accurate and useful [12]. Hence, we use Tsallis entropy to measure the TNF.

Thus, the novel feature of image is extracted now. We put the Tsallis entropy of the TNF into the multi-layer perceptron (MLP). After training, the MLP will automatically classify the images successfully.

## 3. PCNN: A Brief Overview

**Figure 2.**
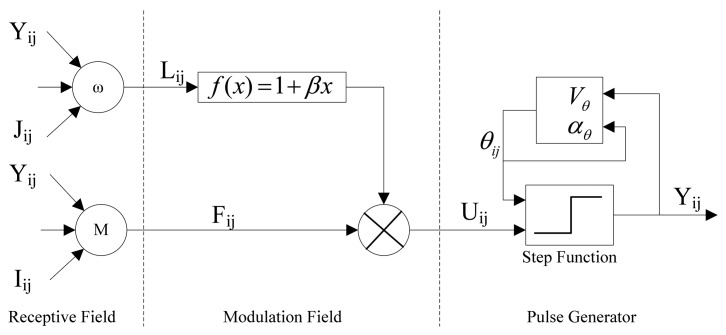
PCNN neuromime.

A typical PCNN neuron consists of three parts: the receptive field, the modulation field and the pulse generator. This is shown in Figure 2.

Suppose *NP* is the total number of iterations and *n* is current iteration, the neuromime of PCNN can be described by the following equations.

(1) $$F_{ij}[n]=\exp(-\alpha_{F})F_{ij}[n-1]+V_{F}\sum m_{\text{ijkl}}Y_{kl}[n-1]+I_{ij}$$

(2) $$L_{ij}[n]=\exp(-\alpha_{L})L_{ij}[n-1]+V_{L}\sum \omega_{\text{ijkl}}Y_{kl}[n-1]$$

(3) $$U_{ij}[n]=F_{ij}[n](1+\beta L_{ji}[n])$$

(4) $$Y_{ij}[n]=\{\begin{array}{cc} 1, & U_{ij}[n]>\theta_{ij}[n-1]\\ 0, & U_{ij}[n]\leq \theta_{ij}[n-1] \end{array}$$

(5) $$\theta_{ij}[n]=\exp(-\alpha_{\theta })\theta_{ij}[n-1]+V_{\theta }Y_{ij}[n-1]$$

where the (*i*, *j*) pairs presents the position of a neuron. *F*, *L*, *U*, *Y* and *θ* are feeding inputs, linking inputs, internal activity, pulse output, and dynamic threshold, respectively. *α_F_*, *α_L_* and *α_θ_* are time constants for feeding, linking and dynamic threshold. *V_F_*, *V_L_* and *V_θ_* are normalizing constants, *M* and *ω* are the synaptic weights, and *I_ij_* and *J_ij_* are external inputs. *β* is the strength of the linking.

Firstly, the neuron (*i*, *j*) receive input signals from other neurons and from external source through the receptive fields. Then the signals are divided into two channels. One is feeding channel (*F*), the other is linking channel (*L*). Secondly, in the modulation part the linking input *L* is weighted with *β* and added a constant bias, then multiplied with the feeding input *F*. The internal activity *U* is the output of the modulation part. Finally, in the pulse generator part *U* compares with the threshold *θ*. If *U* is larger than *θ*, the neuron will emit a pulse. Otherwise it will not emit. Y is the output. The threshold will be adjusted every step. If the neuron has fired, *θ* will increase; otherwise *θ* will decay.

## 4. Obtain TNF via PCNN

There exists a one-to-one correspondence between the image pixels and network neurons, which means, each pixel is associated with a unique neuron and vice versa.

The exponential decay is too time-consuming for fast realization, so an improvement to conventional PCNN is proposed here: the external input *I* and the dynamic threshold *θ* are simplified into corresponding pixel values and an accelerated decrease model, respectively:

(6) $$F_{ij}[n]=I_{ij}$$

(7) $$\theta_{ij}[n]=\{\begin{array}{ccc} \theta_{0}, & n=0,Y[0]=0 & \\ f[n], & Y_{ij}[n-1]=0 & ,\theta_{0}>\theta_{1}\\ +\inf, & Y_{ij}[n-1]=1 & \end{array}$$

where *j* [*n*] presents a monotonically decreased function. The whole process is as follows: the dynamic threshold *θ* descends linearly from its original *θ_1_* to the terminal *θ_0_*, and thus all neurons were initially inhibited (guaranteed by *θ_1_*, meanwhile *Y*=0), and then transformed gradually to be activated. Once a neuron is activated, namely *Y_ij_*=*1*, it will never be activated again (guaranteed by *θ_ij_*=+inf).

During the simulation, each iteration updates the internal activity and the output for every neuron in the network, based on the stimulus signal from the image and the previous state of the network. For each iteration the TNF over the entire PCNN is computed and stored in a global array G. The following describes the details:

Step 1. Initialize the range [*θ_0_, θ_1_*] of dynamic threshold.
Step 2. Simplify the external input *F* into a corresponding gray value *I*, and set the inner linking matrix *W* to a 3 by 3 square matrix with the value of its elements being the reciprocal of square of the Euclidian distance between the central pixel and corresponding pixel.
Step 3. Determine the expression *of f*(*n*) as follows:
  (8) $$f(n)=\frac{(\theta_{1}-\theta_{0})n+\theta_{0}NP-\theta_{1}}{NP-1}$$
  where *NP* presents the whole steps of PCNN which is usually at the range [10, 50].
Step 4. Perform the PCNN. Accumulate *Y* [*i*] and get
  (9) $$S[n]=\sum_{i=1}^{n}Y[i],n=1,2,\text{L},NP$$
  since *Y* [*i*] are not overlapped, *S* [*n*] is also binary. With *n* augmented, the areas of “1” in *S* [*j*] will also be enlarged.
Step 5. Obtain the TNF from *S* [*n*]
  (10) $$G[n]=\sum_{ij}S_{ij}[n],n=1,2,\text{L},NP$$
  *G* [*n*] is then used at next stage of the system.

## 5. The Tsallis Entropy

Entropy is usually used to describe the information contained in the system. Shannon defined the concept of information and described the essential components of a communication system as the following: a sender, a receiver, a communication channel, and an encoding of the information set.

However, communication systems are less well defined when they occur in nature, such as the neurons. Neurons appear to both senders and receivers of information. Another question is what are the communication channels for neurons. They might be the synaptic gaps between the axon and dendrites or not. The answer is not acceptable for neurophysiologists. A more question is what is the encoding of the information. There is no clear and obvious answer.

In general, traditional Shannon entropy is not fit for measuring the information contained by PCNN. Thus, Tsallis entropy *T* is chosen for measuring the information contained in TNF.

(11) $$T=\frac{1-\sum_{i=1}^{N}P^{q}(x_{i})}{q-1}$$

where, *q* is a parameter that is greater than 0. *x* is a random variable with a domain of *N* informational symbols, *x_1_x_2_* L, *x_n_*. Note that, in the limit that *q* goes to 1, this reduces to the standard Boltzman-Gibbs-Shannon measure.

In R. Sneddon's work, he set *q* =2. However, in Section 8 we use an optimization algorithm to computer the optimal value of *q*.

The information contained in TNF is calculated as in the following equation:

(12) $$T[n]=\frac{1-[p^{q}\{G[n]=0\}+p^{q}\{G[n]=1\}}{q-1}$$

where *n* stands for the current iteration.

## 6. The MLP Classifier

**Figure 3.**
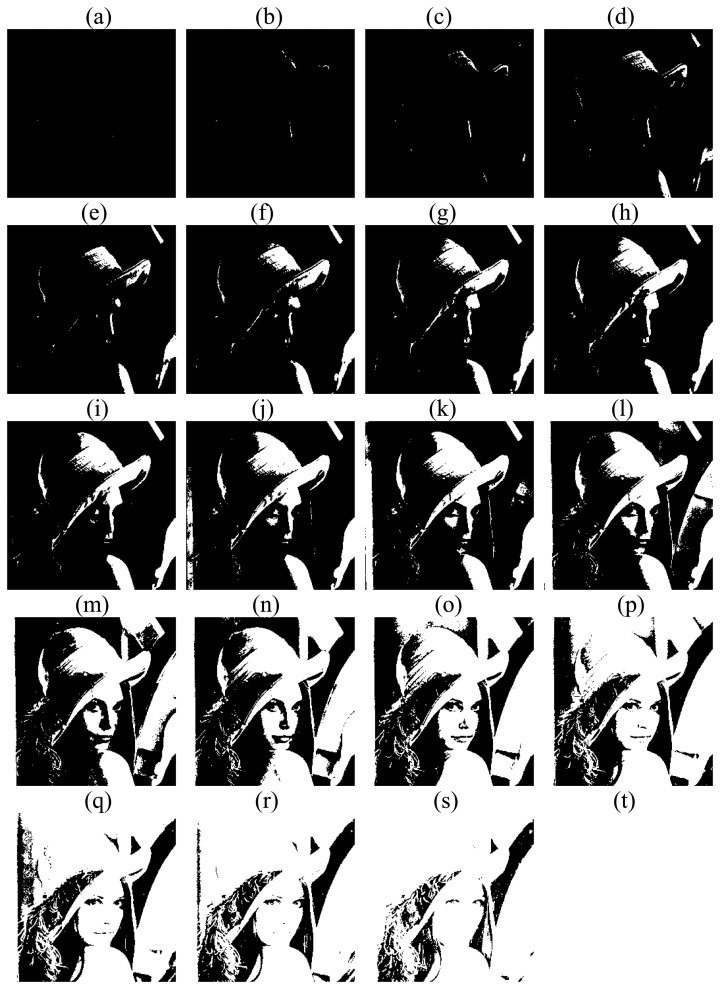
*s* [*n*] of each step of PCNN on Lena. **(a)** Step 1. **(b)** Step 2. **(c)** Step 3. **(d)** Step 4. **(e)** Step 5. **(f)** Step 6. **(g)** Step 7. **(h)** Step 8. **(i)** Step 9. **(j)** Step 10. **(k)** Step 11. **(l)** Step 12. **(m)** Step 13. **(n)** Step 14. **(o)** Step 15. **(p)** Step 16. **(q)** Step 17. **(r)** Step 18. **(s)** Step 19. **(t)** Step 20.

The classifier is basically a MLP. The neural architecture consists of one input layer, one hidden layer and one output neuron (Figure 3). The input layer contains a number of inputs equal to *NP*. Then, the hidden layer has an extension of about 10-20% of the input layer, here we suppose there are *m* neurons at the hidden layer, and the maximum steps is *s*.

Because of the specific task, the output layer contained only one neuron. An output value of 1 is equivalent to target detection whereas a value of 0 means no target detection. A standard back-propagation algorithm is used for supervised training.

## 7. Feature extraction and analysis experiments

The experiments consist of three stages. Firstly we give an example of obtaining Tsallis entropy of TNF. Secondly we check the property of position, scale and rotation independence.

### 7.1. Attaining the Tsallis entropy

Take Lena as an example. First, we normalize the pixel values into the range [0, 1]. Then we perform PCNN with *NP*=*20*. Figure 3 shows *s* [*n*] at each step.

**Figure 4.**
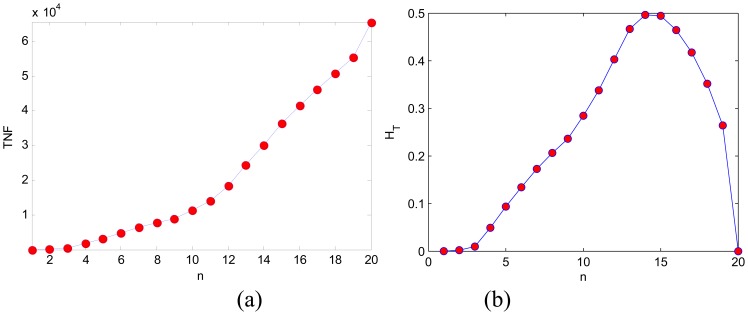
Feature extraction. **(a)***G* [*n*]. **(b)***T* [*n*].

Hence, from Figure 4 it is easy to obtain TNF and the Tsallis entropy, which are shown in Figure 4. The values of TNF vary too large, which is not suitable for directly sended into MLP. And after transforming it to Tsallis entropy, the range of values is compressed to a small interval [0, 0.5].

The *T* [*n*] is not linear with the pattern of Figure 4(a). The maximum point of *T* [*n*] from Figure 4(b) is at 14. Then it is obvious that the 14th subimage in Figure 4 is the most obvious and has the largest contrast. Thus, *T* [*n*] can be understood as the measurement of pattern obviousness.

### 7.2. Translation, Scaling, and Rotation Independence

**Figure 5.**
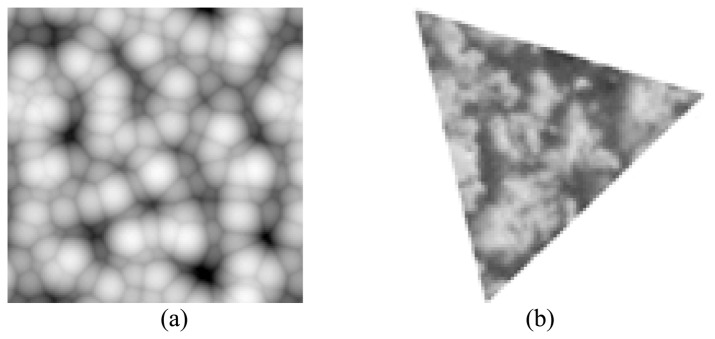
Shapes used for testing. **(a)** A rectangle. **(b)** A triangle.

We use the Tsallis entropy *T* [*n*] as the feature to classify different patterns. At this experiment we will check whether it is a translation, scaling and rotation invariant feature. We use simple geometric shapes as input images, two of them were shown in Figure 5.

**Figure 6.**
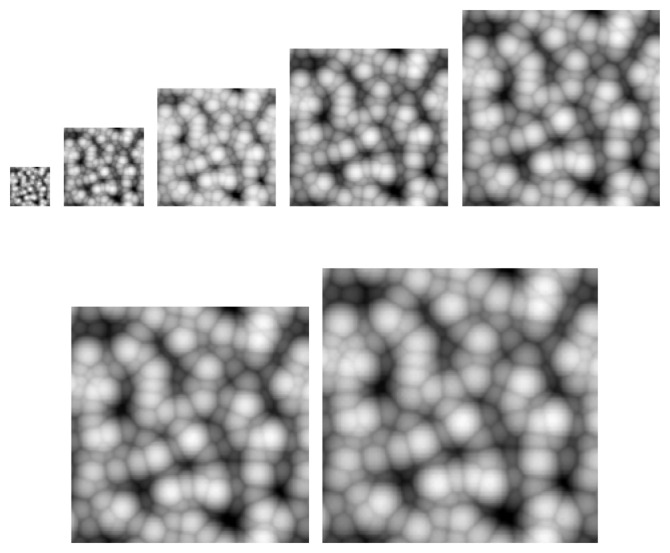
Seven different scales of the rectangle. **(a)** Size=50 pixels. **(b)** Size=100 pixels. **(c)** Size=150 pixels. **(d)** Size=200 pixels. **(e)** Size=250 pixels. **(f)** Size=300 pixels. **(g)** Size=350 pixels.

As expected, the system showed total translation independence. Then seven different scales of the rectangle were selected for testing scaling independence (Figure 6). We find that the *T* [*n*] obtained from these seven scales are quite similar to each other, which demonstrates that this proposed feature is scaling independent. Table 1 shows the feature extracted from different scales of the rectangle. Here MSE is the shortened form of “mean square error”.

**Figure 7.**
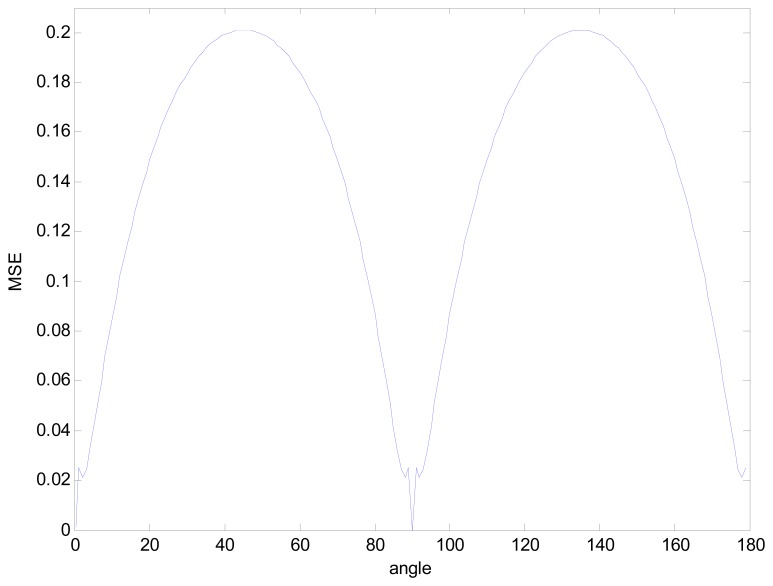
MSE for different rotation angles.

As for the rotation independent, the triangle had been rotated at different angles and the MSE had been computed for each rotation. Results shown in Figure 7 prove that the maximum MSE is obtained for the two principal diagonals (45° and 135°), which seems the rotation independence is a bit weak especially at those two angles, but may be a clue indicating that the MSE is caused by pixel discretization [13]. Test on other hundreds of images also demonstrate the conclusion.

## 8. Face Recognition

**Figure 8.**
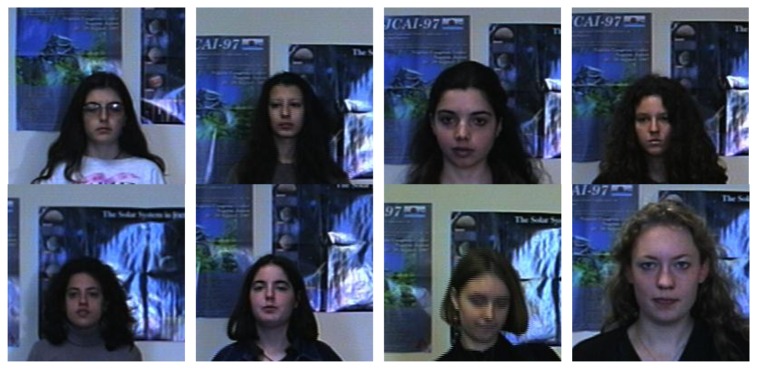
Several typical faces in the database.

From Section 7 it is obvious that the features extracted via our model is effective. Hence, we apply this method to face recognition. The datasets come from the University of Essex School of Computer Science and Electronic Engineering website (http://cswww.essex.ac.uk/mv/allfaces/faces96.html). A sequence of 20 images per individual was taken. During the sequence the subject takes one step forward towards the camera. This movement is used to introduce significant head variations between images of same individual. There is about 0.5 s between successive frames in the sequence. Figure 8 shows several typical faces used in this experiment.

Each individual is averagely split into training and testing sets, namely, 10 images are used for training while the other 10 images are used for testing. The optimal parameters (*q*, *NP*, *m*, *S*) are acquired by the guidance of bacterial chemotaxis optimization (BCO) described in Ref. [14]. The final values are as listed in Table 2.

The highest correct classification rate (CR) of our proposed algorithm has of 72.5%. Although it is not as ideal as some other mature face recognition algorithms, we consider it more potential since research on pattern recognition via PCNN is currently progressing.

**Figure 9.**
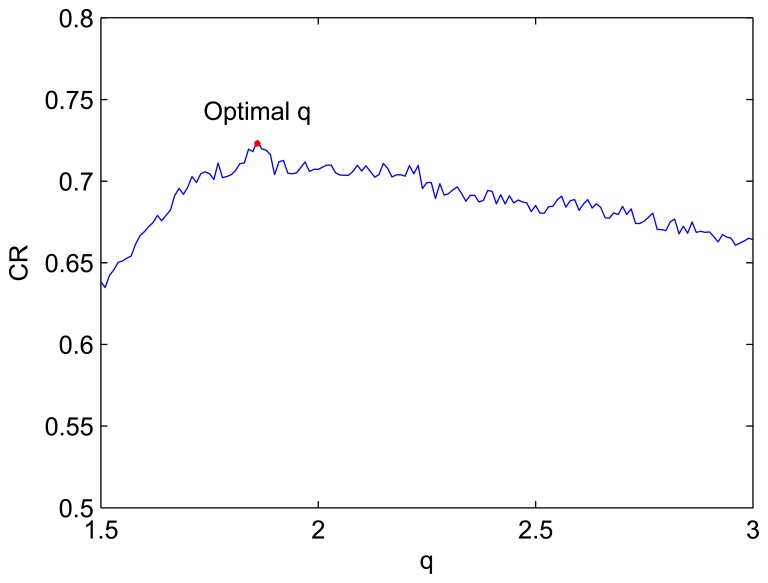
The curve of CR with *q*.

Firstly we adjust one parameter while fix others to check the robustness of our method. Firstly, the parameter *q* is tuned. The result is shown in Figure 9.

From Figure 9, the classification rate remains an acceptable level (>70%) while *q* is in the interval [1.63 - 2.23]. It indicates that the algorithm is robust with *q*. The best value of *q* is 1.86.

**Figure 10.**
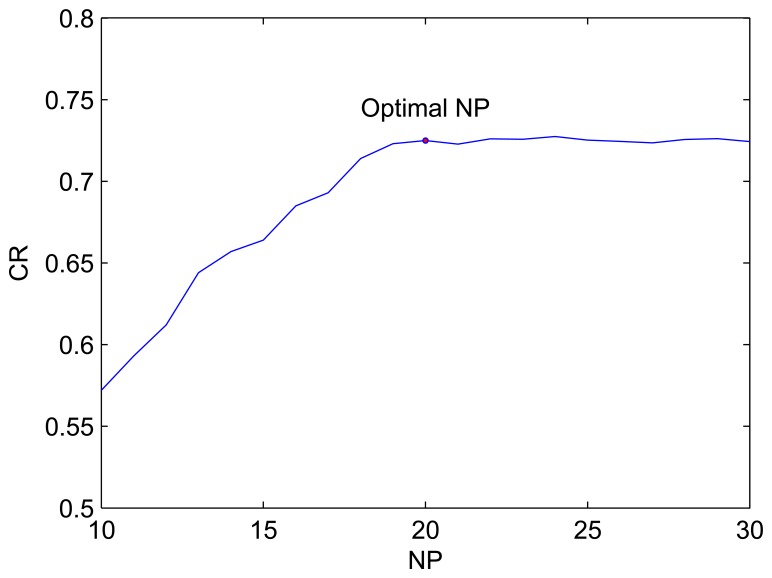
The curve of CR with *NP*.

Secondly, the parameter *NP* was tuned while others remained unchanged. From Figure 10 it is obvious that when *NP* is less than 20, the CR improves with *NP*. As *NP* increases to 20, the CR remains nearly steady. Hence, *NP* is set as 20 taking calculation time into account.

**Figure 11.**
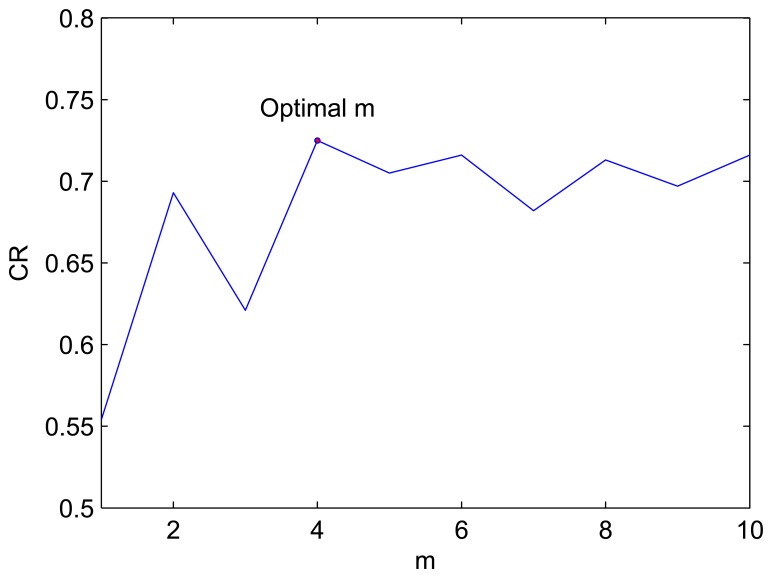
The curve of CR with *m*.

Finally we changed the value of *m*; the results are listed in Figure 11, which implies that the best value of *m* is 4.

The three important parameters are analyzed above. It can conclude that the parameters are essential to the performance of this model. Hence, it is important for researchers to tune these parameters before the network works.

## 9. Conclusion

In this study, a novel feature extraction method was described and applied for face recognition. This paper is just the first attempt to explore the potential of Tsallis entropy of TNF obtained by PCNN to handle the face recognition problems.

Experiments demonstrate that this new feature is unique, translation and scale independent. The future work shall focus on combining this proposed feature with others to improve the classification rate. Another possible research topic is to simplify the procedures in this model. Actually, PCNN does not need pre-training, so the calculation time is expected to decline faster.

Furthermore, this proposed approach is new and potential. It can be applied on all sorts of recognition fields.

## Figures and Tables

**Table 1.** The impact of scaling (Size of the origin image is 200 pixels).

Size	*T* [*n*]	MSE
50	0.0016, 0.0361, 0.1247, 0.2414, 0.3714, 0.4608, 0.4969, 0.4920, 0.4548, 0.3875, 0.3233, 0.2516, 0.1813, 0.1100, 0.0612, 0.0423, 0.0245, 0.0135, 0.0096, 0.0000	3.9524e-3
100	0.0006, 0.0280, 0.1163, 0.2376, 0.3657, 0.4581, 0.4967, 0.4930, 0.4539, 0.3887, 0.3148, 0.2509, 0.1818, 0.1119, 0.0612, 0.0380, 0.0243, 0.0139, 0.0086, 0.0000	1.1543e-3
150	0.0004, 0.0287, 0.1143, 0.2368, 0.3629, 0.4577, 0.4967, 0.4926, 0.4536, 0.3908, 0.3169, 0.2482, 0.1809, 0.1098, 0.0603, 0.0379, 0.0239, 0.0142, 0.0085, 0.0000	7.59e-4
200	0.0006, 0.0288, 0.1167, 0.2371, 0.3634, 0.4579, 0.4968, 0.4925, 0.4533, 0.3913, 0.3162, 0.2480, 0.1818, 0.1106, 0.0618, 0.0377, 0.0238, 0.0141, 0.0090, 0.0000	0
250	0.0004, 0.0285, 0.1165, 0.2381, 0.3635, 0.4583, 0.4967, 0.4926, 0.4539, 0.3921, 0.3167, 0.2484, 0.1815, 0.1110, 0.0629, 0.0378, 0.0242, 0.0142, 0.0088, 0.0000	4.6218e-4
300	0.0007, 0.0290, 0.1168, 0.2384, 0.3638, 0.4582, 0.4967, 0.4926, 0.4538, 0.3923, 0.3165, 0.2487, 0.1817, 0.1116, 0.0627, 0.0377, 0.0240, 0.0143, 0.0090, 0.0000	5.2731e-4
350	0.0006, 0.0294, 0.1168, 0.2389, 0.3639, 0.4584, 0.4967, 0.4925, 0.4537, 0.3923, 0.3163, 0.2482, 0.1819, 0.1120, 0.0631, 0.0379, 0.0242, 0.0145, 0.0090, 0.0000	6.6282e-4

**Table 2.** The optimal parameters used in face recognition.

Parameters	*q*	*NP*	*m*	*S*	*CR*
**Value**	1.86	20	4	50000	72.5%
